# Expression of recombinant 35 kDa fragment of VP2 protein of canine parvovirus using *Escherichia coli* expression system

**DOI:** 10.14202/vetworld.2021.1682-1688

**Published:** 2021-06-29

**Authors:** Natnaree Inthong, Sarawan Kaewmongkol, Nattakan Meekhanon, Eukote Suwan, Wanat Sricharern, Khomson Satchasataporn, Rungthiwa Sinsiri, Kaitkanoke Sirinarumitr, Theerapol Sirinarumitr

**Affiliations:** 1 Center for Agricultural Biotechnology, Kasetsart University, Kamphaeng Saen Campus, Nakhon Pathom 73140, Thailand; 2 Center of Excellence on Agricultural Biotechnology: (AG-BIO/PERDO-CHE), Bangkok 10900, Thailand; 3 Department of Veterinary Technology, Faculty of Veterinary Technology, Kasetsart University, Bangkok 10900, Thailand; 4 Molecular Diagnostic Laboratory, Faculty of Veterinary Medicine, Kasetsart University, Bangkok 10900, Thailand; 5 Department of Companion Animal Clinical Sciences, Faculty of Veterinary Medicine, Kasetsart University, Bangkok 10900, Thailand; 6 Department of Pathology, Faculty of Veterinary Medicine, Kasetsart University, Bangkok 10900, Thailand

**Keywords:** canine parvoviruses, *Escherichia coli* expression system, recombinant protein, *VP2* gene

## Abstract

**Background and Aim:**

Canine parvovirus (CPV) is one of the most common viral infections in dogs, causing acute hemorrhagic gastroenteritis and high mortality. Vaccination effectively prevents CPV infection. However, the currently available CPV vaccines have concerns such as maternal immunity interference, shedding of virus vaccine, and false-positive result based on polymerase chain reaction after vaccination. A subunit vaccine can overcome these problems. This study aimed to express the recombinant 35 kDa fragment of the VP2 protein (consisting of epitopes 1-7) and the recombinant full-length VP2 protein (consisting of epitopes 1-10) and to study the ability of these two recombinant proteins to react with rabbit anti-CPV polyclonal antibodies.

**Materials and Methods:**

The full length and 35 kDa fragment of *VP2* gene of CPV were cloned into the pBAD202 Directional TOPO^™^ expression vector and expressed in *E. coli*. The recombinant full-length and the recombinant 35 kDa fragment proteins of VP2 were analyzed using sodium dodecyl sulfate-polyacrylamide gel electrophoresis and Western blotting.

**Results:**

The recombinant full-length and the recombinant 35 kDa fragment *VP2* genes were successfully cloned and expressed. The optimum concentrations of arabinose and induction time for the recombinant full-length and the recombinant 35 kDa fragment VP2 proteins were 0.2% for 6 h and 0.02% for 6 h, respectively. The recombinant full-length and the recombinant 35 kDa fragment VP2 protein molecular weights were approximately 81 and 51 kDa, respectively. The recombinant full-length and the recombinant 35 kDa fragment VP2 proteins specifically interacted with rabbit anti-CPV polyclonal antibodies.

**Conclusion:**

These results suggest that the recombinant 35 kDa fragment and the recombinant full-length VP2 proteins may be useful in developing a CPV diagnostic test or vaccine.

## Introduction

Canine parvovirus (CPV) is one of the most common viral infections in domestic dogs [1]. It causes acute hemorrhagic gastroenteritis, leukopenia, nausea, diarrhea, and sometimes fatal myocarditis in young puppies [1]. It belongs to the family *Parvoviridae*, subfamily *Parvovirinae*, and the genus *Protoparvovirus* and the species *Carnivore protoparvovirus* 1 [2]. It is a non-enveloped, icosahedral, linearized single-stranded DNA virus. The CPV genome is approximately 5.2 kb in length. Two major open reading frames encode two non-structural proteins (NS1 and NS2) and three structural proteins (VP1, VP2, and VP3). The non-structural proteins are required for DNA transcription and replication. The VP1 and VP2 proteins are constructed by alternative splicing of the similar messenger RNA. The VP3 protein is formed by cleavage from the VP2 protein terminus [3-5].

There are two types of commercial vaccines that prevent CPV infection: the inactivated and modified live virus vaccines. The inactivated vaccine is less immunologically protective than the modified live vaccine and requires a booster several times to achieve immunity that can protect against CPV infection [6]. The modified live virus vaccine is more effective in reducing CPV infection [7]. However, the modified live virus vaccine can infect, replicate in enterocytes, and produce long-term viremia in the vaccinated dog. Furthermore, the long-term shedding of the virus vaccine in the feces of a vaccinated dog may interfere with diagnostic tests and cause reinfection in the animal [8].

The VP2 capsid protein is the main capsid protein, having a molecular weight of 65 kDa and consisting of 584 amino acid residues [9,10]. The VP2 protein plays a key role in inducing neutralizing antibodies and CPVs host range. By PEPSCAN and mutation analyses, 10 epitopes have been identified in two neutralizing antigenic sites (A and B) of the VP2 protein on the CPV surface [11,12]. The A site consists of loops 1, 2, and 4. Epitopes 1-4 are located on threefold spikes of loop 1. Epitope 5 is situated on loop 2 [7,12]. The B site consists of loop 3 with epitopes 6 and 7 located on the shoulder of the 3-fold spike [3,7,12,13]. High antigenicity of CPVs VP2 protein has been reported on loops 1 and 3 that included epitopes 1-7 [12,14].

A subunit vaccine is a vaccine that presents one or more antigens to the immune system without introducing pathogen particles, whole or otherwise. The subunit vaccine offers several advantages over the conventional vaccine, such as no vaccine breakdown, no virus vaccine shedding, and the capability to distinguish the vaccine from the field strain virus [15-17]. A prokaryotic expression system has the advantages of a high yield and ease of producing recombinant proteins [15,16].

This study aimed to express the recombinant 35 kDa fragment of the VP2 protein (consisting of epitopes 1-7) and the recombinant full-length VP2 protein (consisting of epitopes 1-10) and to study the ability of these two recombinant proteins to react with rabbit anti-CPV polyclonal antibodies.

## Materials and Methods

### Ethical approval

This study was approved by the Animal Ethics Committee of the Faculty of Veterinary Medicine, Kasetsart University, Bangkok, Thailand (ACKU62-VET-007).

### Study period and location

The study was conducted during Febuary 2019-January 2020 at the Faculty of Veterinary Medicine and the Faculty of Veterinary Technology, Kasetsart University, Thailand.

### Amplification of full-length and 35 kDa fragment *VP2* gene of CPV

The plasmid containing the full-length *VP2* gene (accession number KP715680) from a previous study [18] was used as a template for amplifying both the full-length and the 35 kDa fragment *VP2* genes. The primer sets for amplifying the full-length and the 35 kDa fragment *VP2* genes are shown in Table-1. The CACC sequence at the 5’ end of the forward primer for amplifying both the full-length and the 35 kDa fragment *VP2* genes was added because it is required for cloning into the pBAD202/D TOPO® vector (Invitrogen, Carlsbad, CA, USA), following the manufacturer’s instructions. The polymerase chain reaction (PCR) mixture for both the full-length and the 35 kDa fragment *VP2* genes was composed of 20 μL of 5× Phusion HF Buffer, 2 μL of 10 mM dNTPs, 1 μL of each forward and reverse primers, 1 μL of Phusion High-Fidelity DNA Polymerase (Thermo Scientific, Waltham, MA, USA), 10 μL of template DNA, and 65 μL of distilled water to produce a total volume of 100 μL. The PCR conditions were pre-denaturation at 98°C for 30 s, followed by 35 cycles of denaturation at 98°C for 10 s, annealing at 48°C (for the full-length VP2) or 51°C (for the 35 kDa fragment of VP2) for 30 s, extension at 72°C for 45 s, and a final extension at 72°C for 10 min. The expected PCR products of the full-length and the 35 kDa fragment of *VP2* gene were 1752 and 951 bps, respectively.

**Table-1:** Primers used for cloning of full-length and 35 kDa fragment of *VP2* gene.

VP2	Antigenic sites	Primer sequence (5’-3’)	PCR products (bps)	Predicted molecular weight of VP2 protein (kDa)
35 kDa fragment	1-7	F=CACC ATG AGT GAT GGA GCA GTT CA	951	~35
		R=AGT TAC ACC ACG TCT TTT ATC TTG TTG		
Full length	1-10	F=CACC ATG AGT GAT GGA GCA GTT CA	1752	~65
		R=ATA TAA TTT TCT AGG TGC TAG TTG		

### Cloning of full-length and 35 kDa fragment *VP2* gene of CPV-2

One hundred microliters of PCR products of each segment were purified using an UltraClean^®^15DNA purification kit (MO BIO Laboratories, Inc., Carlsbad, CA, USA), and 20 μL of PCR products of each segment were eluted. The eluted PCR products of each segment were used to clone into plasmid pBAD202/D-TOPO (pBAD Directional TOPO^®^ Expression Kit; Invitrogen, Carlsbad, CA, USA), following the manufacturer’s protocol. Briefly, the purified PCR products of each segment were mixed with 1 μL of salt solution and 1 μL of the pBAD202/D-TOPO^®^ vector. The ligation mixture was mixed and incubated at 23°C for 1 h. Six microliters of the ligation mixture were used to transform into TOP10 competent cells (Invitrogen, Carlsbad, CA, USA). Subsequently, 900 μL of SOC medium (pBAD Directional TOPO^®^ Expression Kit; Invitrogen) were added into the transformed TOP10 competent cells and shaken at 37°C for 1 h. Subsequently, transformed TOP10 competent cells (pBAD Directional TOPO^®^ Expression Kit; Invitrogen) were spread on Luria-Bertani (LB) agar containing 50 mg/mL of kanamycin. The positive colonies were confirmed using PCR, and the PCR products from positive colonies were submitted for sequencing (First BASE Laboratories Sdn Bhd, Selangor, Malaysia).

### Optimization conditions for induction of recombinant full-length and recombinant 35 kDa fragment proteins of *VP2* gene

The inocula were prepared by adding a single colony of each recombinant *Escherichia coli* into 2 mL of LB broth containing 50 mg/mL of kanamycin and shaken at 200 rpm at 37°C overnight. Then, 100 mL of each inoculum were added into six tubes containing 10 mL of LB broth with 50 mg/mL kanamycin and shaken for 3 h or until the optical density (OD_600_) reached a value of approximately 0.5. The 10-fold serial dilution (final concentrations of 0.2%, 0.02%, 0.002%, 0.0002%, 0.00002%, and 0%) of stock arabinose solution (20%) was added to LB broth for the induction of each inoculum. The cultures were collected 4 h after induction and kept at −80°C for further verification using sodium dodecyl sulfate-polyacrylamide gel electrophoresis (SDS-PAGE). For the optimum induction time of recombinant proteins, 10 mL of the cultures were induced using 0.2% arabinose (for the recombinant full-length VP2 protein) or 0.02% arabinose (for the recombinant 35 kDa fragment of VP2 protein); harvested every 2 h at 0, 2, 4, 6, and 8 h after induction; and kept at −80°C for further verification using SDS-PAGE. The TOP10 competent cells without the recombinant plasmids were used as a negative control.

### SDS-PAGE

One hundred microliters of lysis buffer were added to the induced recombinant *E. coli* pellet and boiled for 15 min. The extracted protein of each segment was measured using a NANODROP 2000c spectrophotometer (Thermo Scientific, Waltham, MA, USA). Then, 160 mg of extracted proteins were loaded into each lane of 10% SDS-PAGE gel and electrophoresed for 50 min at 150 V. Gels were stained with staining solution (0.01% Coomassie Brilliant Blue, 50% distilled water, 40% methanol, and 10% acetic acid) for 30 min and subsequently destained with destaining solution (50% distilled water, 40% methanol, and 10% acetic acid) twice for 30 min or until the band of protein appeared. The recombinant proteins were then purified using Ni-NTA columns (Ni Sepharose^™^ 6 Fast Flow, GE Healthcare Bio-Sciences AB, Uppsala, Sweden) following the manufacturer’s instructions.

### Preparation of polyclonal antibodies against CPV

The polyclonal antibodies against CPV were prepared by immunizing three New Zealand white rabbits with 1 mL of CPV modified live vaccine (Primodog^™^, Merial, Lyon, France) at weeks 0, 2, 4, and 6. Five to ten milliliters of blood were collected 2 weeks before and after the booster. Sera were collected using low-speed centrifugation, aliquoted, and kept at −20°C until used.

### Western blot (WB) analysis

Each sample of crude and purified recombinant full-length and the recombinant 35 kDa fragment of VP2 proteins was separated using 10% SDS-PAGE gel and electrotransferred onto a nitrocellulose membrane at 10 V and 400 mA for 45 min. The nitrocellulose membranes were blocked with 5% skim milk at 4°C overnight. Then, the membranes were incubated with either 1:10,000 rabbit anti-histidine polyclonal antibodies (6x-His Tag Polyclonal Antibody PA1-983B; Thermo Fisher Scientific, MA, USA) or 1:100 rabbit anti-CPV polyclonal antibodies at 25°C for 1 h. Subsequently, the membranes were washed 3 times with PBS buffer containing 0.1% Tween 20 (1×PBS-T). After washing 3 times with 1× PBS-T for 10 min each, the membranes were incubated with 1:2000 goat anti-rabbit IgG conjugated with horseradish peroxidase (SeraCare, Milford, MA, USA) for 1 h at 37°C. After washing, the membranes were incubated with diaminobenzidine (DAB) using a DAB substrate kit (Thermo Fisher Scientific, MA, USA) for 5-10 min at 25°C. Each recombinant VP2 protein was visualized as a brown band on the nitrocellulose membrane.

## Results

**Figure-1:**
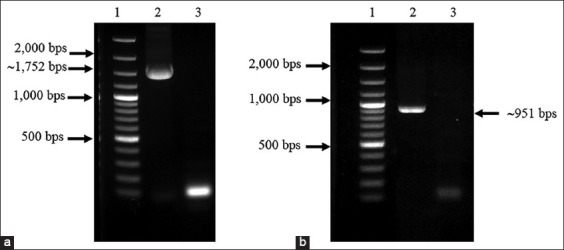
About 1% agarose gel electrophoresis of PCR products of positive clone for full-length (a) and 35 kDa fragment of *VP2* gene (b). Lane 1 = GeneRuler 100 bp Plus DNA ladder (Thermo Scientific); lane 2 = PCR products of full length of *VP2* gene (a) or 35 kDa fragment of *VP2* gene (b); and lane 3 = negative control.

**Figure-2:**
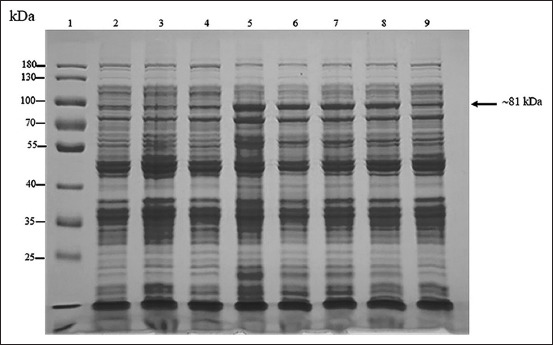
About 10% sodium dodecyl sulfate-polyacrylamide gel electrophoresis analysis of optimum concentration of arabinose for induction of recombinant full length of VP2 protein (~81 kDa). Lane 1 = PageRuler™ Plus Prestained Protein Ladder (Thermo Scientific); lanes 2 and 3 = proteins from wild-type Top 10 competent cells induced by 0.2% arabinose at 0 and 4 h, respectively; lane 4 = proteins from recombinant Top 10 competent cells containing full-length *VP2* gene induced by 0.2% arabinose at 0 h; and lanes 5-9 = proteins from recombinant Top 10 competent cells containing full-length *VP2* gene induced by 0.2, 0.02, 0.002, 0.0002, and 0.00002% arabinose concentration at 4 h after induction, respectively.

**Figure-3:**
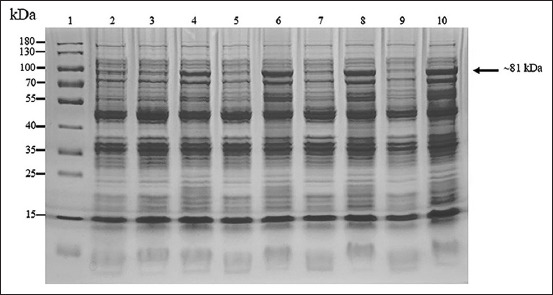
About 10% sodium dodecyl sulfate-polyacrylamide gel electrophoresis analysis of optimum time for induction of recombinant full length of VP2 protein (~81 kDa). Lane 1 = PageRuler™ Plus Prestained Protein Ladder (Thermo Scientific); lanes 2, 3, 5, 7, and 9 = protein from wild-type Top 10 competent cells induced by 0.2% arabinose at 0, 2, 4, 6, and 8 h, respectively; and lanes 4, 6, 8, and 10 = protein from the recombinant Top 10 competent cells containing full-length *VP2* gene induced by 0.2% arabinose at 2, 4, 6, and 8 h, respectively.

**Figure-4:**
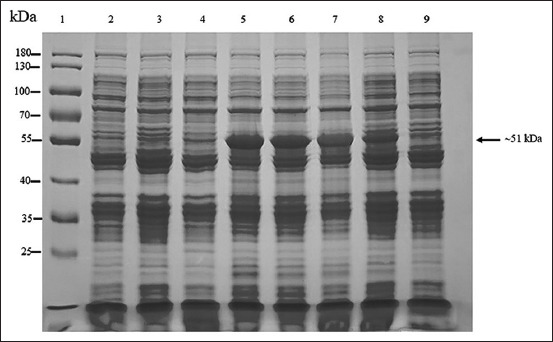
About 10% sodium dodecyl sulfate-polyacrylamide gel electrophoresis analysis of optimum concentration of arabinose for induction of recombinant 35 kDa of VP2 protein (~51 kDa). Lane 1 = PageRuler™ Plus Prestained Protein Ladder (Thermo Scientific), lanes 2 and 3 = proteins from wild-type Top 10 competent cells induced by 0.2% arabinose at 0 and 4 h, respectively; lane 4 = proteins from recombinant Top 10 competent cells containing 35 kDa fragment of *VP2* gene induced by 0.2% arabinose at 0 h; and lanes 5-9 = proteins from recombinant Top 10 competent cells containing 35 kDa fragment of *VP2* gene induced by 0.2, 0.02, 0.002, 0.0002, and 0.00002% arabinose concentration at 4 h after induction, respectively.

**Figure-5:**
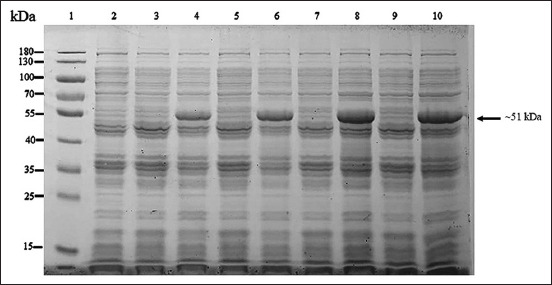
About 10% sodium dodecyl sulfate-polyacrylamide gel electrophoresis analysis of optimum time for induction of recombinant 35 kDa of VP2 protein (~51 kDa). Lane 1 = PageRuler™ Plus Prestained Protein Ladder (Thermo Scientific); lanes 2, 3, 5, 7, and 9 = proteins from wild-type Top 10 competent cells induced by 0.2% arabinose at 0, 2, 4, 6, and 8 h, respectively; and lanes 4, 6, 8, and 10 = proteins from the recombinant Top 10 competent cells containing 35 kDa fragment of *VP2* gene induced by 0.2% arabinose at 2, 4, 6, and 8 h, respectively.

**Figure-6:**
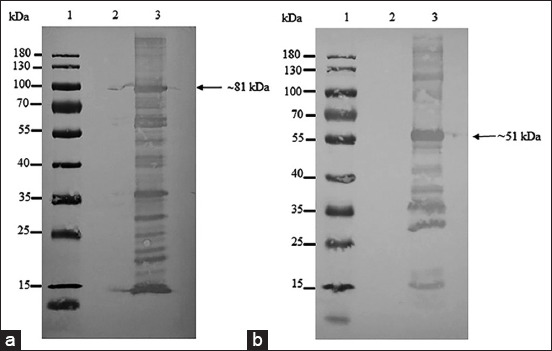
Western blot analysis of the recombinant crude full-length (a) and the recombinant crude 35 kDa fragment proteins (b) of VP2 using 1:10,000 rabbit anti-histidine polyclonal antibodies. Lane 1 = PageRuler™ Plus Prestained Protein Ladder (Thermo Scientific); lane 2 = crude protein from wild-type Top 10 competent cells; and lane 3 = recombinant crude full length of VP2 protein (a) and recombinant crude 35 kDa fragment of VP2 protein (b).

**Figure-7:**
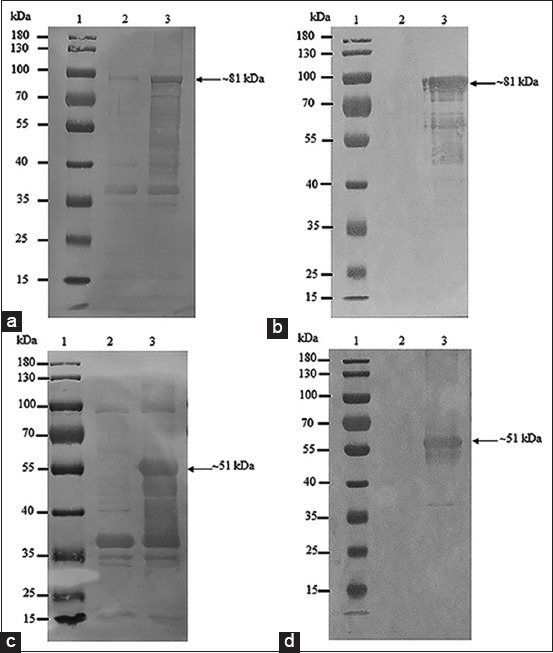
Western blot analysis of recombinant crude (a and c) and purified (b and d) full-length (a and b) and 35 kDa fragment proteins of VP2 (c and d) using 1:100 rabbit anti-CPV polyclonal antibodies. Lane 1 = PageRuler™ Plus Prestained Protein Ladder (Thermo Scientific); lane 2 = crude (a and c) and purified (b and d) proteins from wild-type Top 10 competent cells; and lane 3 = recombinant crude full length of VP2 protein (a), recombinant purified full length of VP2 protein (b), recombinant crude 35 kDa fragment of VP2 protein (c), and recombinant purified 35 kDa fragment of VP2 protein (d).

The amplification of the full-length and 35 kDa fragment *VP2* genes was successful. The PCR products of the full-length (1752 bps) and the 35 kDa fragment *VP2* gene (951 bps) were used to ligate with the pBAD202/D-TOPO vector. The positive clones were confirmed using PCR (Figure-1), and the correctness of inserted genes was confirmed based on sequencing. The optimum conditions for the expression of the recombinant full-length and the recombinant 35 kDa fragment VP2 proteins were 0.2% arabinose for 6 h (Figures-2 and 3) and 0.02% arabinose for 6 h (Figures-4 and 5), respectively. The molecular weights of the recombinant full-length and the recombinant 35 kDa fragment VP2 proteins were approximately 81 and 51 kDa, respectively. Both the recombinant full-length and the recombinant 35 kDa fragment VP2 proteins were insoluble. Based on WB analysis, the samples of crude and purified recombinant full-length and 35 kDa fragment VP2 proteins showed positive reaction with rabbit anti-histidine polyclonal antibodies (Figure-6) and rabbit anti-CPV polyclonal antibodies (Figure-7).

## Discussion

The recombinant full-length and the recombinant 35 kDa fragment proteins of the *VP2* gene were successfully cloned and expressed. The optimum conditions for the expression of recombinant full-length and 35 kDa fragment proteins of *VP2* gene were determined. The molecular weights of the two proteins were established, and for both recombinant proteins, they were 16 kDa higher than expected due to the thioredoxin and 6×histidine included in the plasmid pBAD 202/D-TOPO.

There have been several studies on the expression of either recombinant full-length or partial VP2 proteins of CPV, such as in mammalian cells [10,19], insect cells [20,21], and *E. coli* system [7,9,22]. The recombinant full-length and 35 kDa fragment VP2 proteins in the present study were insoluble, as observed in other studies that used *E. coli* as the VP2 expression host [9,22]. However, this had no effect on the ability of the recombinant VP2 protein to interact with polyclonal antibodies against CPV [9,22-24], which was similar to the present study. Insoluble recombinant proteins are generated due to incorrectly folded proteins and stabilized by removing hydrophobic residues [25]. The conformational epitope of the recombinant protein might not be detected by antibody due to the incorrect folding. Linear epitopes might be preferred for applications in which the protein target is wholly or partially denatured during the sample preparation before the immunoassay, such as in WB, immunohistochemistry, or immunofluorescence-based confocal microscopy [26]. All 10 epitopes of VP2 protein were detected by PEPSCAN analysis [11,12], implying that these epitopes might be linear. Thus, the recombinant full-length and the recombinant 35 kDa fragment VP2 proteins in this study detected by polyclonal antibodies against CPV may be due to detecting linear epitopes presenting on the VP2 protein.

In this study, the recombinant full-length and the 35 kDa fragment VP2 proteins interacted specifically with rabbit anti-CPV polyclonal antibodies, even though there are 10 antigenic sites in the recombinant full-length VP2 protein and only seven antigenic sites in the recombinant 35 kDa fragment VP2 protein; this may have been due to the high immunoactivity of the VP2 protein of CPV located on loops 1 and 3 [12,14] that contain epitopes 1-7. In addition, the recombinant truncated VP2 protein containing epitope 5 or epitope 6-7 expression in *E. coli* has been shown to interact with canine serum vaccinated with CPV vaccine [24]. These recombinant truncated VP2 proteins are good candidates for ELISA or latex agglutination tests for CPV. Moreover, antigenic sites 6-7 have been reported as candidate antigenic sites to develop a subunit vaccine [23].

## Conclusion

The recombinant 35 kDa fragment and the recombinant full-length VP2 proteins reacted with rabbit anti-CPV polyclonal antibodies. Based on the current results, *E. coli* expression system showed promise to express either the recombinant full-length or the recombinant 35 kDa fragment VP2 proteins. The expression system is easy to implement, is inexpensive, and has a high-protein yield. The recombinant 35 kDa fragment VP2 protein might be a good candidate for developing a diagnostic test for differentiating infected from vaccinated animal vaccine for CPV.

## Authors’ Contributions

NI and TS: Designed the experiment, conducted all experiments, and wrote the manuscript. SK, NM, ES, WS, KS, RS, KaS, and TS: involved in scientific discussion and provided suggestions for the overall work. All authors have read and approved the final manuscript.

## References

[1] Prittie J (2004). Canine parvoviral enteritis:A review of diagnosis, management, and prevention. J. Vet. Emerg. Crit. Care.

[2] Ogbu K.I, Mira F, Purpari G, Nwosuh C, Loria G.R, Schirò G, Chiaramonte G, Tion M.T, Di Bella S, Ventriglia G, Decaro N, Anene B.M, Guercio A (2020). Nearly full-length genome characterization of Canine parvovirus strains circulating in Nigeria. Transbound. Emerg. Dis.

[3] MacLachlan N.J, Dubovi E.J, MacLachlan N.J, Dubovi E.J (2016). Parvoviridae. Fenner's Veterinary Virology.

[4] Payne S, Payne S (2017). Family parvoviridae. Viruses.

[5] Miranda C, Thompson G (2016). Canine parvovirus:The worldwide occurrence of antigenic variants. J. Gen. Virol.

[6] Vartak A, Sucheck S.J (2016). Recent advances in subunit vaccine carriers. Vaccines (*Basel*).

[7] Xu J, Guo H.C, Wei Y.Q, Dong H, Han S.C, Ao D, Sun D.H, Wang H.M, Cao S.Z, Sun S.Q (2014). Self-assembly of virus-like particles of Canine parvovirus capsid protein expressed from *Escherichia coli*and application as virus-like particle vaccine. Appl. Microbiol. Biotechnol.

[8] Decaro N, Crescenzo G, Desario C, Cavalli A, Losurdo M, Colaianni M.L, Ventrella G, Rizzi S, Aulicino S, Lucente M.S, Buonavoglia C (2014). Long-term viremia and fecal shedding in pups after modified-live Canine parvovirus vaccination. Vaccine.

[9] Park J.S, Choi B.K, Vijayachandran L.S, Ayyappan V, Chong C.K, Lee K.S, Kim S.C, Choi C.W (2007). Immunodetection of Canine parvovirus (CPV) in clinical samples by polyclonal antisera against CPV-VP2 protein expressed in *Escherichia coli* as an antigen. J. Virol. Methods.

[10] Gupta P.K, Rai A, Rai N, Raut A.A, Chauhan S (2005). Cloning of Canine parvovirus VP2 gene and its use as DNA vaccine in dogs. Curr. Sci.

[11] Wikoff W.R, Wang G, Parrish C.R, Cheng R.H, Strassheim M.L, Baker T.S, Rossmann M.G (1994). The structure of a neutralized virus:Canine parvovirus complexed with neutralizing antibody fragment. Structure.

[12] Langeveld J.P, Casal J.I, Vela C, Dalsgaard K, Smale S.H, Puijk W.C, Meloen R.H (1993). B-cell epitopes of canine parvovirus:Distribution on the primary structure and exposure on the viral surface. J. Virol.

[13] Llamas-Saiz A.L, Agbandje-McKenna M, Parker J.S, Wahid A.T, Parrish C.R, Rossmann M.G (1996). Structural analysis of a mutation in Canine parvovirus which controls antigenicity and host range. Virolog.

[14] Feng H, Liang M, Wang H.L, Zhang T, Zhao P.S, Shen X.J, Zhang R.Z, Hu G.Q, Gao Y.W, Wang C.Y, Wang T.C, Zhang W, Yang S.T, Xia X.Z (2011). Recombinant canine parvovirus-like particles express foreign epitopes in silkworm pupae. Vet. Microbiol.

[15] Cheng L.T, Chung Y.C, Yang C.D, Chuang K, Ke G.M, Chu C.Y, Gopalakrishnakone P (2015). Animal vaccine technology:An overview. Toxinology:Biological Toxins and Bioterrorism.

[16] Gomes A, Byregowda S, Veeregowda B, Vinayagamurthy B (2016). An overview of heterologous expression host systems for the production of recombinant proteins. Adv. Anim. Vet. Sci.

[17] Wang M, Jiang S, Wang Y (2016). Recent advances in the production of recombinant subunit vaccines in *Pichia pastoris*. Bioengineered.

[18] Inthong N, Kaewmongkol S, Meekhanon N, Sirinarumitr K, Sirinarumitr T (2020). Dynamic evolution of Canine parvovirus in Thailand. Vet. World.

[19] Luo J, Shi H, Tan Y, Niu X, Long T, Zhao J, Tian Q, Wang Y, Chen H, Guo X (2016). Two potential recombinant rabies vaccines expressing Canine parvovirus virion protein 2 induce immunogenicity to Canine parvovirus and rabies virus. Vaccine.

[20] Jiao C, Zhang H, Liu W, Jin H, Liu D, Zhao J, Feng N, Zhang C, Shi J (2020). Construction and immunogenicity of virus-like particles of feline parvovirus from the tiger. Viruses.

[21] Jin H, Xia X, Liu B, Fu Y, Chen X, Wang H, Xia Z (2016). High-yield production of Canine parvovirus virus-like particles in a baculovirus expression system. Arch. Virol.

[22] Phromnoi S, Sinsiri R, Sirinarumitr T (2010). Expression of recombinant VP2 protein of Canine parvovirus in *Escherichia coli*. Kasetsart J. Nat. Sci.

[23] Thomas J, Singh M, Goswami T.K, Glora P, Chakravarti S, Chander V, Upmanyu V, Verma S, Sharma C, Mahendran K (2017). Determination of immune status in dogs against CPV-2 by recombinant protein based latex agglutination test. Biologicals.

[24] Lijun S, Jing W, Peng W, Gang L, Miaomiao G, Weifeng Y, Hongfei Z (2012). Development of an indirect enzyme-linked immunosorbent assay (ELISA) assay based on a recombinant truncated VP2 (tVP2) protein for the detection of Canine parvovirus antibodies. Afr. J. Biotechnol.

[25] Foo D.G.W, Ang R.X, Alonso S, Chow V.T.K, Quak S.H, Poh C.L (2008). Identification of immunodominant VP1 linear epitope of enterovirus 71 (EV71) using synthetic peptides for detecting human anti-EV71 IgG antibodies in western blots. Clin. Microbiol. Infect.

[26] Forsström B, Axnäs B.B, Rockberg J, Danielsson H, Bohlin A, Uhlen M (2015). Dissecting antibodies with regards to linear and conformational epitopes. PloS One.

